# Insights in the maturational processes influencing hydrocortisone pharmacokinetics in congenital adrenal hyperplasia patients using a middle-out approach

**DOI:** 10.3389/fphar.2022.1090554

**Published:** 2023-01-12

**Authors:** Robin Michelet, Davide Bindellini, Johanna Melin, Uta Neumann, Oliver Blankenstein, Wilhelm Huisinga, Trevor N. Johnson, Martin J. Whitaker, Richard Ross, Charlotte Kloft

**Affiliations:** ^1^ Department of Clinical Pharmacy and Biochemistry, Institute of Pharmacy, Freie Universitaet Berlin, Berlin, Germany; ^2^ Graduate Research Training Program, Berlin, Germany; ^3^ Clinic for Pediatric Endocrinology and Diabetology, Charité-Universitätsmedizin, Berlin, Germany; ^4^ Institute of Mathematics, Universität Potsdam, Potsdam, Germany; ^5^ Certara UK Limited, Simcyp Division, Sheffield, United Kingdom; ^6^ Department of Oncology and Metabolism, University of Sheffield, Sheffield, United Kingdom; ^7^ Diurnal Limited, Cardiff, United Kingdom

**Keywords:** hydrocortisone, congenital adrenal hyperplasia, population pharmacokinetics, middle-out approach, pediatrics, physiologically-based pharmacokinetics (PBPK), non-linear mixed effects modelling (NLME), maturation

## Abstract

**Introduction:** Hydrocortisone is the standard of care in cortisol replacement therapy for congenital adrenal hyperplasia patients. Challenges in mimicking cortisol circadian rhythm and dosing individualization can be overcome by the support of mathematical modelling. Previously, a non-linear mixed-effects (NLME) model was developed based on clinical hydrocortisone pharmacokinetic (PK) pediatric and adult data. Additionally, a physiologically-based pharmacokinetic (PBPK) model was developed for adults and a pediatric model was obtained using maturation functions for relevant processes. In this work, a middle-out approach was applied. The aim was to investigate whether PBPK-derived maturation functions could provide a better description of hydrocortisone PK inter-individual variability when implemented in the NLME framework, with the goal of providing better individual predictions towards precision dosing at the patient level.

**Methods:** Hydrocortisone PK data from 24 adrenal insufficiency pediatric patients and 30 adult healthy volunteers were used for NLME model development, while the PBPK model and maturation functions of clearance and cortisol binding globulin (CBG) were developed based on previous studies published in the literature.

**Results:** Clearance (CL) estimates from both approaches were similar for children older than 1 year (CL/F increasing from around 150 L/h to 500 L/h), while CBG concentrations differed across the whole age range (CBG_NLME_ stable around 0.5 μM vs. steady increase from 0.35 to 0.8 μM for CBG _PBPK_). PBPK-derived maturation functions were subsequently included in the NLME model. After inclusion of the maturation functions, none, a part of, or all parameters were re-estimated. However, the inclusion of CL and/or CBG maturation functions in the NLME model did not result in improved model performance for the CL maturation function (ΔOFV > −15.36) and the re-estimation of parameters using the CBG maturation function most often led to unstable models or individual CL prediction bias.

**Discussion:** Three explanations for the observed discrepancies could be postulated, i) non-considered maturation of processes such as absorption or first-pass effect, ii) lack of patients between 1 and 12 months, iii) lack of correction of PBPK CL maturation functions derived from urinary concentration ratio data for the renal function relative to adults. These should be investigated in the future to determine how NLME and PBPK methods can work towards deriving insights into pediatric hydrocortisone PK.

## 1 Introduction

Congenital adrenal hyperplasia (CAH), a disease which leads to very low to no cortisol synthesis, is the commonest cause of adrenal deficiency in childhood. Patients have an increased morbidity and mortality in adult life that may in part relate to suboptimal glucocorticoid therapy in their early years of life (Finkielstain et al., 2012; Han et al., 2014; Bancos et al., 2015; Bornstein et al., 2016). Lifelong glucocorticoid replacement therapy with hydrocortisone is standard of care for CAH patients and personalized replacement therapy through precision medicine is essential in optimizing care (Merke and Bornstein, 2005; Hindmarsh, 2009; Kamoun et al., 2013). Hydrocortisone, which is chemically the same as endogenous cortisol, is administered multiple times per day due to its short terminal half-life and to approximate the physiological cortisol circadian rhythm (Knutson et al., 1997; Hindmarsh and Charmandari, 2015). Therefore, treating pediatricians are constantly faced with the risk of over- and under-dosing their patients, which may lead to complications of excess steroid therapy (Falhammar et al., 2014) and adrenal crisis (El-Maouche et al., 2018), respectively.

Mathematical models to investigate and quantify the sources of intra- and inter-individual variability (IIV) in pharmacokinetics (PK) and pharmacodynamics (PD) of drugs can help to support the choice of the right dose at the right time for the right patient in the form of model-informed precision dosing (Kluwe et al., 2020). This approach would be of value in helping optimize and individualize hydrocortisone replacement in neonates, infants and older children with CAH. To do this, a mathematical model needs to be able to describe the underlying processes in sufficient detail to capture the succinct parts while still being able to quantify and explain sources of variability to apply model predictions at the individual level. For the individualization of hydrocortisone treatment in (especially young) children, this means foremost an acceptable characterization of the PK of this endogenous compound across the pediatric age range.

Recently, both non-linear mixed effects (NLME) modelling of clinical data (the so-called ‘top-down’ approach) and physiologically-based pharmacokinetic (PBPK) modelling (the so-called ‘bottom-up’ approach) were applied to describe the PK of hydrocortisone (Melin et al., 2017; Michelet et al., 2020; Bonner et al., 2021). In the first approach, the authors were able to use clinical pediatric hydrocortisone PK data from CAH patients in combination with adult data to inform an NLME model quantifying the IIV in hydrocortisone PK across the pediatric age range (Melin et al., 2017), which was then optimized and used to simulate possible optimized dosing regimens (Michelet et al., 2020). However, the available clinical data was too sparse to quantify an impact of age on the PK parameters after taking body weight into consideration. In the second approach, a PBPK model was developed and qualified for hydrocortisone PK in adults, which was then combined with ontogeny functions obtained from literature data for the relevant processes to obtain a pediatric PBPK model (Bonner et al., 2021). These ontogeny functions focused on the maturation of 5α-reductase, 11-β hydroxysteroid dehydrogenase 2 (11-βHSD2), and cortisol binding globulin (CBG), known to be influential on hydrocortisone PK (Hadjian et al., 1975; Walker and Seckl, 2003; Wudy et al., 2007). A third approach to describe PK variability based on both aforementioned approaches, the so-called ‘middle-out’ approach was recently applied in pediatric PK modeling as combining the ‘best-of-two-worlds’ but has thus far not been applied to pediatric hydrocortisone PK (Tsamandouras et al., 2013; Michelet et al., 2018a; Michelet et al., 2018b; Germovsek et al., 2018). The benefit of this approach would be that the physiological insights coming from a PBPK approach could be implemented within the hierarchical variability quantification framework of an NLME approach, allowing for individual predictions of hydrocortisone PK and the application of stochastic simulations for evaluation of personalized dosing strategies.

In this manuscript, we investigated whether using such a middle-out approach by implementing the PBPK-derived insights regarding maturation of hydrocortisone PK processes into an NLME-framework based on available clinical data could better describe the interindividual variability of hydrocortisone PK, paving the way for model-based precision medicine dosing of hydrocortisone, particularly in pediatric CAH patients.

## 2 Methods

### 2.1 Patient characteristics and study design

The patient populations used in this work has been described elsewhere (Melin et al., 2017; Melin et al., 2020; Michelet et al., 2020). In short, for the pediatric patients, cortisol concentrations were collected in an open label, phase 3, single center clinical trial conducted at the Institute of Experimental Paediatric Endocrinology at Charité-Universitätsmedizin Berlin, CVK, Berlin (EudraCT number: 2014–002265-30). Written informed consent was given by parents/guardian and the study was approved by the relevant independent ethics committee (Ethics committee of Berlin, No. 14/0517- EK 12). Paediatric patients with adrenal insufficiency (23 with congenital adrenal hyperplasia and 1 with hypopituitarism) aged from birth to 6 years were included. One dose of individualized hydrocortisone granules (Alkindi^®^, Diurnal Europe B.V., Netherlands), corresponding to the individual standard morning dose (1–4 mg) was administered in the morning upon arrival to the clinic after at least 2 h fasting. Patients were not allowed to eat within 60 min post-dose (30 min for children below 1 year). All patients underwent plasma sampling prior to dose, 1 and 4 h post-dose. Three additional samples were retrieved per patient in cohort 1 (1–6 years), every individual was randomized into one of four groups (*n* = 3) in which two extra samples were taken after approximately 30, 45, 90, 120, 150, and/or 180 min and for all an extra sample around the expected minimum concentration (T_min_) was taken.

For the adult healthy volunteers, data from two independent crossover studies (Infacort-001 and Infacort-002; EudraCT Number: 2013–000260-28 and EudraCT Number: 2013–000259-42) were included. For the arms considered in this work, healthy males between 18 and 60 years were included and received either single morning oral doses of 0.5, 2, 5, and 10 mg of individualized hydrocortisone granules (study 1, *n* = 16) or a single dose of 20 mg individualized hydrocortisone granules (study 2, *n* = 14). For both groups, dexamethasone (1 mg) was administered to suppress the endogenous cortisol synthesis. In study 1, plasma samples were taken at pre-dose and 0.5, 1, 1.5, 2.5, 3, 3.5, 4, 4.5, 5, 5.5, 6, 6.5, 7, 7.5, 8, 9, 10, 11, and 12 h post-dose and cortisol total concentrations were measured. In study 2, total cortisol and CBG were measured in plasma pre-dose, and 0.25, 0.5, 0.75, 1, 1.25, 1.5, 2, 2.5, 3, 4, 5, 6, 8, 10, and 12 h post-dose/period start.

### 2.2 Modelling approaches

#### 2.2.1 Non-linear mixed-effects model

The previously developed NLME model was a semi-mechanistic model based on adult total and unbound hydrocortisone concentrations, allometrically scaled to the pediatric population. The model was further optimized based on the aforementioned data derived from a clinical trial using a pediatric formulation of hydrocortisone that allows accurate dosing in neonates, infants and children with adrenal insufficiency. Using the pediatric body weight and CBG, the semi-mechanistic PK model established on adult data could relatively well predict the observed pediatric observations. However, observed pre-dose concentrations in the pediatric CAH patients were often much higher than predicted by the cortisol baseline based on dexamethasone-suppressed adults. This discrepancy was hypothesized to result from the not-considered maturation of the enzyme 11-βHSD2, causing cyclic resynthesis from cortisone to cortisol (Martinerie et al., 2012). However, estimating the parameters of a semi-mechanistic PK model including maturation of this enzyme was not supported by the sparse pediatric data set, so an allometrically scaled model with separate baselines based on the adult and pediatric datasets was proposed as final model, resulting in good parameter precision for both fixed-effect and variability parameters. In this model, neonates had a lower and more variable relative clearance (CL per kg body weight) than infants, young children and adults, which can potentially be explained by the lower activity of 11-βHSD2 (converting cortisol to cortisone) (Martinerie et al., 2012) and 5α-reductase (irreversible metabolism of cortisol to 5α-DHF (allodihydrocortisol)) (Thigpen et al., 1993) in this age group. Conversely, relative CL in infants was predicted to be more variable than in children and adults, potentially due to the high activity of 5α-reductase in infants relative to their body size and incomplete maturation processes.

#### 2.2.2 Physiologically-based pharmacokinetic model

The previously developed PBPK model (Bonner et al., 2021) was constructed as follows: published adult studies describing the PK of intravenous (IV) hydrocortisone (Toothaker and Welling, 1982; Derendorf et al., 1991) were used to establish the initial drug parameters for distribution and elimination. The volume of distribution was described using a minimal PBPK model, this is akin to a 2-compartmental PK model plus liver compartment, this model also allowed the simulation of changing fraction unbound (f_u_) on volume of distribution. The f_u_ was simulated based on binding to both albumin and CBG, protein reference values and dissociation constants are detailed in the original publication (Bonner et al., 2021). Cortisol elimination input intrinsic clearance values for 11-βHSD2, 5α-reductase, CYP3A4 and additional CL (lumped 20β-oxoreductase and 5β-reductase pathways) were calculated using a retrograde model based on an IV clearance of 20 L/h and the literature derived fraction eliminated by each pathway of 30, 31.5, 2.5%, and 36%, respectively, Data from published studies describing immediate-release oral hydrocortisone PK (Toothaker et al., 1982; Derendorf et al., 1991) were then used to establish the absorption model parameters for the immediate-release formulations of hydrocortisone, and to provide further verification of the model. Once developed, clinical studies of the immediate-release multi-particulate formulation in adults (Infacort-001 and Infacort-002) were used to verify the final model before performing simulations in the paediatric population.

The Sim-Paediatric population was used for the latter which considers the relevant developmental physiology including the ontogeny of albumin and CYP3A4 expression (Johnson et al., 2006). For this study, further information was included on the ontogeny of CBG (meta-analysis of multiple sources including (Hadjian et al., 1975)), 11-βHSD2 and 5α-reductase. The ontogeny of 11β-HSD2 was derived based on urinary cortisone to cortisol ratios (Rogers et al., 2014), and that for 5α-reductase from urinary allo-tetrahydrocortisol/tetrahydrocortisol ratios (meta-analysis of multiple sources including (Wudy et al., 2007), equations are below. The final model was able to capture the majority of clinical data for the ages 2 to 4.7, 0.3 to 1.8, and 0.044–0.071 years within the 5th and 95th percentiles for the simulations.

#### 2.2.3 Middle-out approach

As the maturation of different enzymatic processes was already hypothesized during the development of the NLME model, and formalized during the development of the PBPK model, the next step was to implement the PBPK-derived maturation functions into the NLME model. For this, the maturation of the CL and plasma-protein binding related processes identified in the PBPK model were considered: 5α-reductase (Equation ((1), (1) and (2)1-βHSD2 (Eq. 3) and CBG (Eq. (4)). Similar as in the PBPK model, CYP3A4-related metabolism was assumed to mature rapidly from birth onwards and, due to its contribution to the metabolism of 2.5%, assumed to have a negligible impact on the total CL (Kearns et al., 2003; Hines, 2007).

$$5\alpha -reductase\;\left(0-0.25\;y\right)=0.05+\left(\frac{\left(14.82-0.05\right)*{AGE}^{1.17}}{0.17^{1.17}+{AGE}^{1.17}}\right)$$ (1)

$$5\alpha -reductase\;\left(>0.25\;y\right)=1.56+\left(9.22*\;e^{-1.78*\left(AGE-0.16\right)}\right)$$ (2)

$$11-\beta HSD2=0.02+\left(\frac{\left(1.52-0.02\right)*{AGE}^{0.15}}{0.27^{0.15}+{AGE}^{0.15}}\right)$$ (3)

The PBPK-derived maturation functions for the binding to CBG was considered as it was originally derived (Bonner et al., 2021):

$$CBG\;\left(µM\right)=0.195+\left(\frac{\left(0.993-0.195\right)*{AGE}^{0.348}}{2.33^{0.348}+{AGE}^{0.348}}\right)$$ (4)

Further details regarding Equations 1–4 can be found in the original work describing the development of the hydrocortisone PBPK model (Bonner et al., 2021).

The PBPK-derived maturation functions for the metabolic enzymes were combined in a CL maturation function Eq. 5 following their proportional impact on the overall hydrocortisone CL based on the PBPK-derived contribution to the metabolism: 30% for 5α-reductase, 30% for 11-βHSD2 and 40% for other processes assumed not to undergo relevant maturation (Bonner et al., 2021).

$${CL}_{ratio\frac{pediatric}{adult}}\left(AGE\right)=30\%*{5\alpha -reductase}_{AGE}+30\%*\;{11-\beta HSD2}_{AGE}+40\%$$ (5)

In order to investigate the utility of these maturation functions in the middle-out NLME framework, a step-wise approach was taken.

1) Comparison of maturation function-derived CL and CBG concentration to NLME-derived empirical Bayes estimates (EBE): CL_EBE_ and CBG_EBE._
2) Implementation of CL or CBG maturation functions in NLME model and re-estimation of none, parts of and full model. The procedure was performed for both maturation functions separately and then for both together.
3) Implementation of best-performing maturation functions into NLME model.

For step 1, CL_EBE_ were compared to CL values derived from PBPK maturation function Eq. 5. For this, the age-dependent CL-ratio calculated by the maturation function was multiplied with the adult population CL/F value estimated by the NLME model and corrected for body weight using allometric scaling as shown in Eq. 6.

$${CL}_{PBPK}={CL}_{NLME,\;adult}*{CL}_{ratio\frac{pediatric}{adult}}\left(AGE\right)*\;{\left(\frac{BW\left(kg\right)}{70\;kg}\right)}^{0.75}$$ (6)

To lessen the impact of allometric scaling assumptions and bioavailability, the elimination half-lives, defined as Eq. 7 for a two-compartmental model, were also calculated and compared.

$$t_{\frac{1}{2},\;\beta }=\frac{\ln\left(2\right)}{0.5*\left(\frac{Q}{V_{1}}+\frac{Q}{V_{2}}+\frac{CL}{V_{1}}-\sqrt{{\left(\frac{Q}{V_{1}}+\frac{Q}{V_{2}}+\frac{CL}{V_{1}}\right)}^{2}-4*\frac{Q}{V_{2}}*\frac{CL}{V_{1}}}\right)}$$ (7)

In step 2), PBPK-derived maturation functions were included in the NLME model separately and then both at the same time. Under all three scenarios, model performance was evaluated following one of four estimation steps. First, the parameters from the NLME model and the PBPK-derived maturation functions were not re-estimated and the model was evaluated as such. Second, the NLME-derived PK parameters were re-estimated while keeping the PBPK-derived maturation parameters fixed. Third, the PBPK-derived maturation parameters were re-estimated while keeping the NLME-derived PK parameters fixed. Fourth and final, all parameters were re-estimated based on the clinical dataset.

In general, model evaluation was performed based on predictive performance assessed by goodness-of-fit (GOF) plots and model stability assessed by condition number and parameter precision. Significant differences in model fit were defined as the difference in objective function value (OFV) being larger than 3.84*n_parameters estimated_ (*p* < 0.05). To avoid bias in residuals calculated based on the M3 method, distributions of Normalized Prediction Distribution Errors (NPDEs (Brendel et al., 2006)) rather than Conditionally Weighted Residuals (CWRES) as a function of time and population prediction were used to judge the model fit (Jaber et al., 2021). Individual clearance estimates using empirical Bayes estimates were plotted as a function of age group to investigate introduction of age-dependent bias into the model. All estimations were performed using the FOCE + I algorithm.

### 2.3 Software

Data handling and management were performed using R/RStudio (version 4.0.1/1.3.1056), as well as data visualization. Modelling activities in the middle-out NLME framework were performed using NONMEM (version 7.5.0) and Pearl speaks NONMEM (PsN, version 5.0.0).

## 3 Results

### 3.1 Comparison of individual parameters derived by PBPK and NLME approach

**FIGURE 1:**
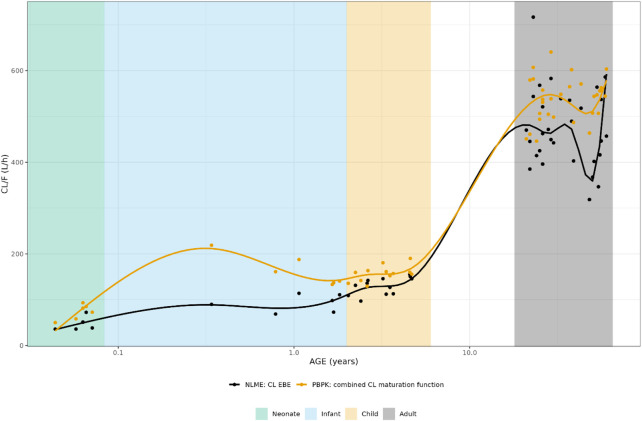
Comparison of individual apparent hydrocortisone clearance for neonates, infants, children and adults after administration of a pediatric formulation of hydrocortisone. The closed circles represent the individual values taking body weight (non-linear mixed effects model) or body weight and age (maturation function) into account. The lines represent Loess smoothers through the individual values. Age shown on a logarithmic scale.

**FIGURE 2:**
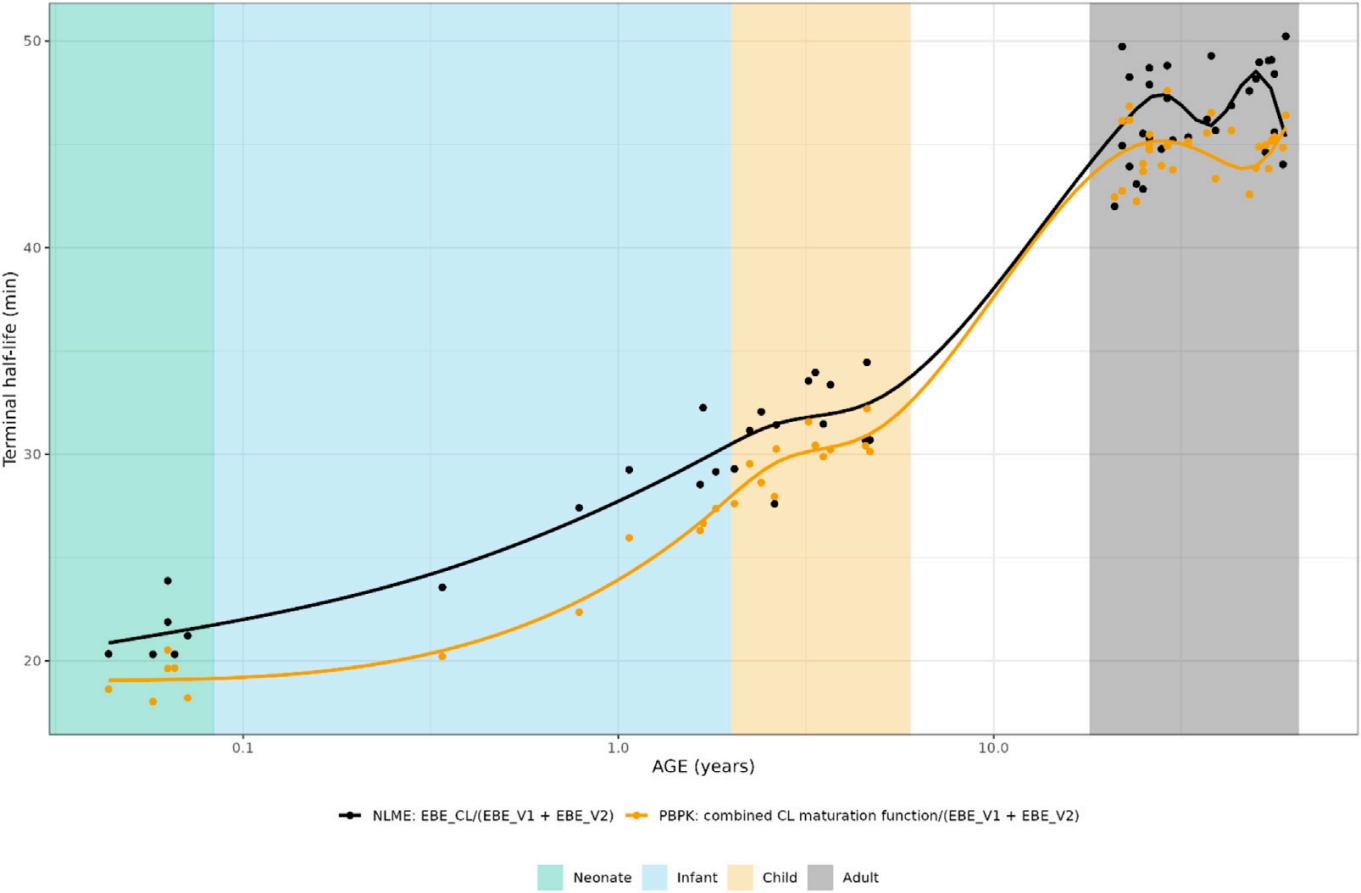
Comparison of individual hydrocortisone terminal half-life for neonates, infants, children and adults after administration of a pediatric formulation of hydrocortisone. The closed circles represent the individual values taking body weight (non-linear mixed effects model) or body weight and age (maturation function) into account. The lines represent Loess smoothers through the individual values. Age shown on a logarithmic scale.

The individually predicted CL and elimination half-lives are presented in Figure 1 and Figure 2. A relatively large overlap could be observed for children and adults older than 1 year. However, for children younger than 1-year substantial discrepancies in predicted elimination processes between the approaches were shown, possibly indicating the relevance of maturational processes in this age range.

For CBG, the PBPK-derived concentrations could be directly compared to the NLME derived ones. The NLME model was parametrized in a way that when a CBG measurement was available for an individual, this measurement was used in the model. When such a measurement was not available, the mean CBG concentration (22.4 μg/mL/0.431 µM) from an earlier developed CBG binding model was used (Melin et al., 2019), which was only the case for 16 adult individuals.

**FIGURE 3:**
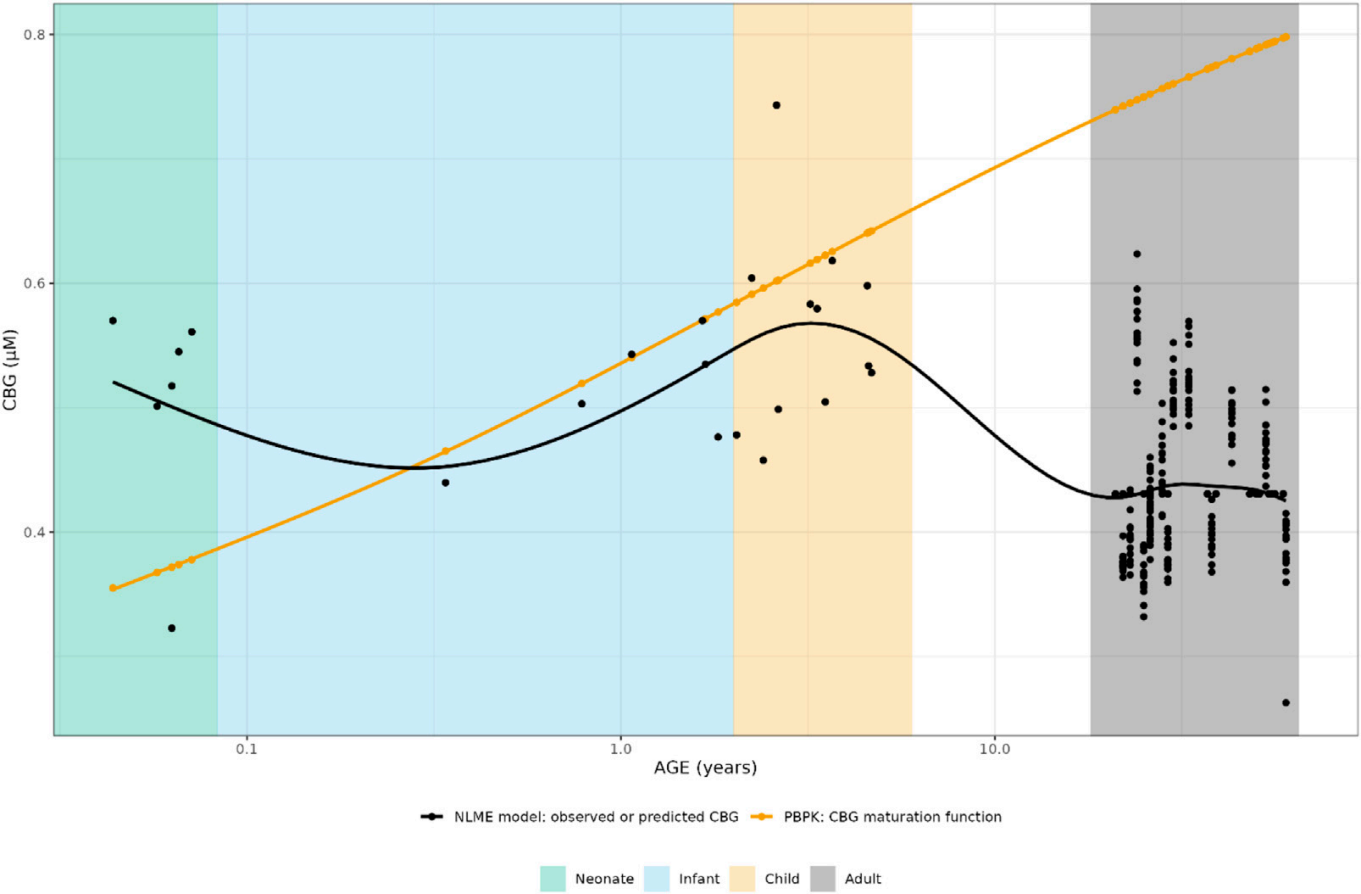
Comparison of individual cortisol-binding globulin concentrations for neonates, infants, children and adults. The closed circles represent the individual values taking body weight (non-linear mixed effects model) or body weight and age (maturation function) into account. The lines represent Loess smoothers through the individual values. Age shown on a logarithmic scale.

As can be seen in Figure 3, the PBPK-derived maturation in CBG concentrations was not represented in the NLME-based approach, resulting in a discrepancy over almost the entire age range.

### 3.2 Implementation of CL or CBG maturation functions in NLME model

To investigate whether the discrepancies in individual parameters between the NLME and PBPK approach had a significant impact on the description of the observed clinical data and as such could provide a possibility for model improvement using a middle-out approach, the implementation of the PBPK-derived maturation functions within the NLME modelling framework was carried out.

#### 3.2.1 Implementation of CL maturation

**FIGURE 4:**
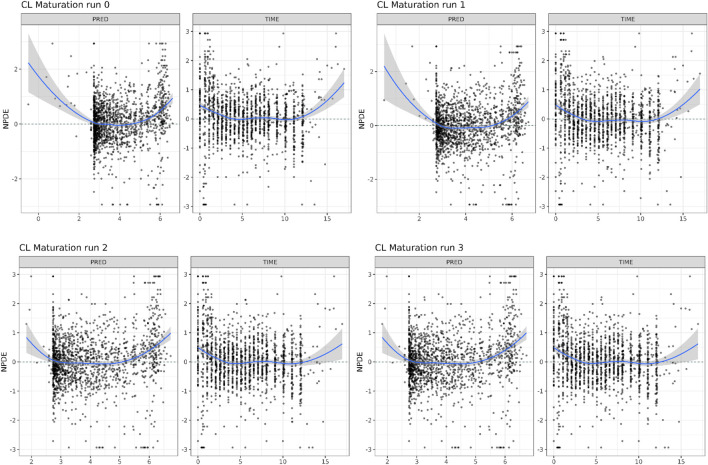
Normalized Distribution Prediction Errors (NPDE) of the 4 middle-out models incorporating the PBPK-derived clearance maturation function. Run 0: no re-estimation of parameters, run 1: re-estimation of NLME-derived PK parameters, run 2: re-estimation of PBPK-derived clearance maturation function, run 3: re-estimation of all parameters.

In Table 1, the parameter estimates of the implemented CL maturation functions within the NLME model are show. Inclusion of the maturation function without re-estimation of any parameter but including fitting of the individual profiles using empirical Bayes estimates (option MAXEVAL = 0 in NONMEM) resulted in a significantly worse fit as indicated by the OFV (ΔOFV = 34.48). Estimation of the PK parameters while keeping the maturation function constant also resulted in a worse fit (ΔOFV = 13.25) and in an unstable model as indicated by failure in convergence of the covariance matrix. Estimating the parameters of the maturation function alone or together with the PK parameters resulted in a non-significant improvement of the fit (ΔOFV = −9.22 > -15.36 for run 2 and −9.34 > −46.08 for run 3), also indicating by the NPDE distributions in Figure 4.

**TABLE 1:** Parameter estimates, objective function value (OFV) and condition number (CN) from the middle-out models exploring the inclusion of a maturation function for cortisol clearance (CL).

Model	Base model	CL-maturation, no re-estimation run 0	CL-maturation, PK-estimation run 1	CL-maturation, MAT-estimation run 2	CL-maturation, re-estimation all’ run 3
Parameter	Value (rse, %)	Value (rse, %)	Value (rse, %)	Value (rse, %)	Value (rse, %)
CL [L/h]	410 (8.1)	409 (-)	325 (ND)	409 (-)	484 (ND)
V2 [L]	10.6 (9.4)	10.6 (-)	10.4 (ND)	10.6 (-)	10.6 (ND)
Q [L/h]	160 (17.9)	160 (-)	147 (ND)	160 (-)	162 (ND)
V3 [L]	124 (16.3)	124 (-)	122 (ND)	124 (-)	127 (ND)
Km [nmol]	4,810 (21.2)	4,810 (-)	5,190 (ND)	4,810 (-)	4,830 (ND)
Vmax [nmol/h]	21,600 (11.0)	21,600 (-)	21,500 (ND)	21,600 (-)	21,600 (ND)
BASEAdult	15.4 (6.33)	15.4 (-)	15.4 (6.33)	15.4 (-)	15.4 (ND)
BASEChild	13.3 (1.94)	13.3 (-)	13.3 (1.94)	13.3 (-)	13.3 (ND)
IIVCL (CV%)	19.2 (17.8)	19.2 (-)	22.9 (ND)	17.2 (ND)	17.3 (ND)
IIV_Km_ (CV%)	45.6 (36.3)	45.6 (-)	40.7 (ND)	43.0 (ND)	42.9 (ND)
IIV_Vmax_ (CV%)	43.7 (16.7)	43.7 (-)	42.9 (ND)	43.0 (ND)	43.0 (ND)
IIV_BASE_ (CV%)	33.5 (22.3)	33.5 (-)	33.5 (ND)	33.5 (ND)	33.5 (ND)
IIV_BIO_ (CV%)	34.9 (19.3)	34.9 (-)	36.5 (ND)	35.2 (ND)	35.2 (ND)
BASE_1,5A_		0.05 (-)	0.05 (-)	0.04 (ND)	0.003 (ND)
MAX_1,5A_		14.8 (-)	14.8 (-)	3.24 (ND)	2.54 (ND)
HILL_5A_		1.17 (-)	1.17 (-)	1.45 (ND)	1.66 (ND)
TM_50,5A_		0.17 (-)	0.17 (-)	0.04 (ND)	0.04 (ND)
BASE_2,5A_		1.56 (-)	1.56 (-)	1.51 (ND)	1.10 (ND)
MAX_2,5A_		9.22 (-)	9.22 (-)	51.1 (ND)	310 (ND)
DEC_5A_		1.78 (-)	1.78 (-)	59.8 (ND)	110 (ND)
INFP_5A_		0.16 (-)	0.16 (-)	0.29 (ND)	0.29 (ND)
BASE_11B_		0.02 (-)	0.02 (-)	0.01 (ND)	0.002 (ND)
MAX_11B_		1.52 (-)	1.52 (-)	0.58 (ND)	0.50 (ND)
HILL_11B_		0.15 (-)	0.15 (-)	143 (ND)	173 (ND)
TM_50,11B_		0.27 (-)	0.27 (-)	4.05 (ND)	4.38 (ND)
Residual variability (CV%)	14.5 (8.0)	14.5 (-)	14.5 (ND)	14.5 (ND)	14.5 (ND)
Condition number	155.6	NA	ND	ND	ND
OFV	-3,907.90	-3,838.94	-3,894.65	-3,917.12	-3,917.24

CL: Apparent clearance, V2: apparent central volume of distribution, Q: apparent intercompartmental clearance, V3: peripheral volume of distribution, K_m_: amount in depot compartment resulting in half of V_max_, V_max_: maximum absorption rate, BASE_Adult_: cortisol baseline of dexamethasone suppressed healthy adults, BASE_Child_: cortisol baseline of children with baseline measurement BLOQ, IIV: interindividual variability, BASE_1,5A_: 5-alpha reductase activity at birth, MAX_1,5A_: maximum 5-alpha reductase activity during first 3 months of life, HILL_5A_, hill factor for the 5-alpha reductase ontogeny function during the first 3 months of life, TM_50,5A_: age at which half of MAX_1,5A_ is reached, BASE_2,5A_, 5-alpha reductase activity at 3 months of age, MAX_2,5A_: maximum 5-alpha reductase activity after first 3 months of life, DEC_5A_: 5-alpha reductase activity decay rate, INFP_5A_: inflection point of the 5-alpha reductase activity ontogeny function, BASE_11B_: 11-β hydroxysteroid dehydrogenase 2 activity at birth, MAX_11B_: maximum 11-β hydroxysteroid dehydrogenase 2 activity during life, HILL_11B_: hill factor for the 11-β hydroxysteroid dehydrogenase 2 ontogeny function, TM_50,11B_: age at which half of MAX_11B_ is reached. Parameters were allometrically scaled using a body weight of 70 kg and residual variability was estimated as additive error on a log scale.

**FIGURE 5:**
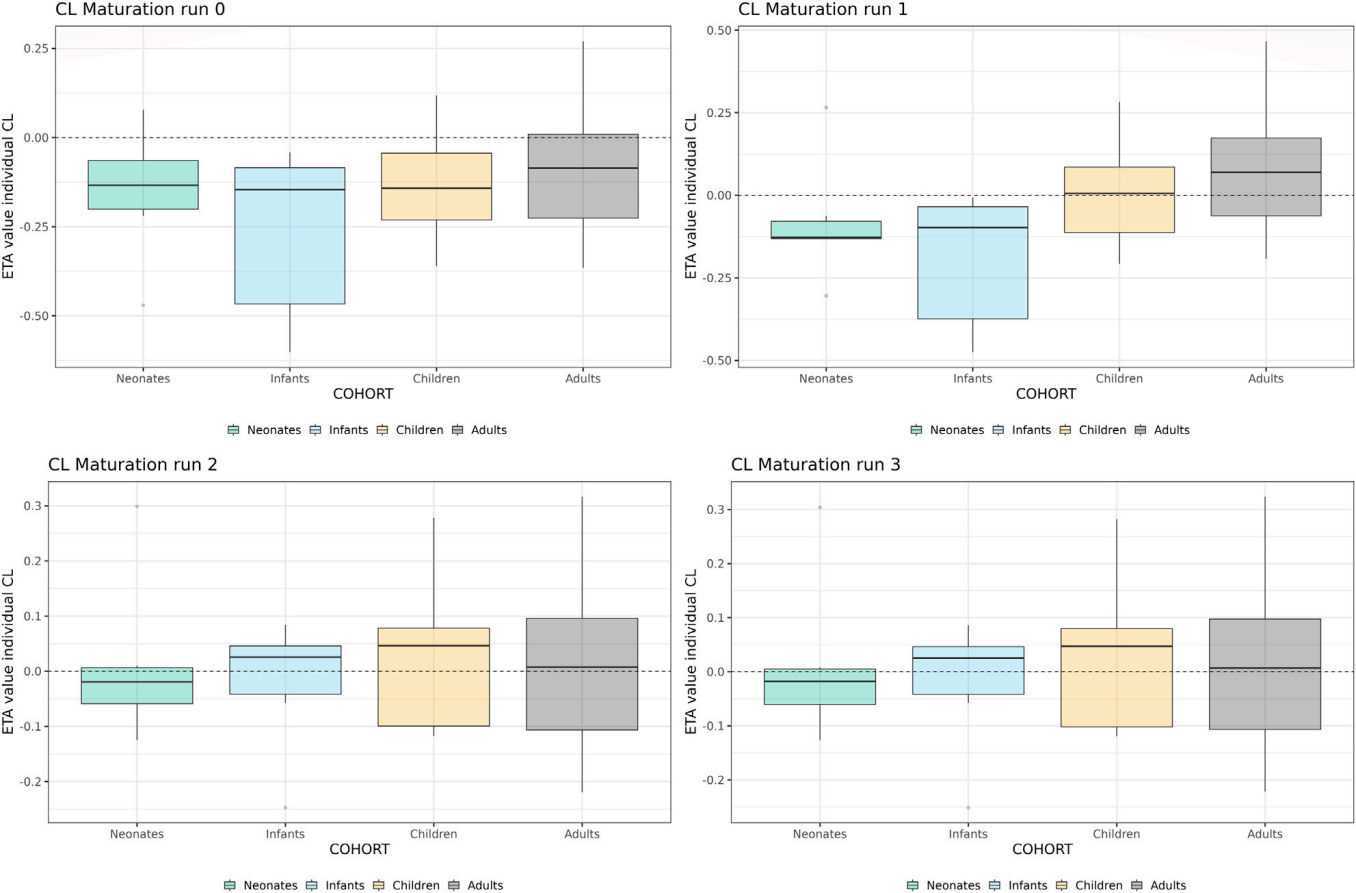
Individual clearance parameter estimates (empirical Bayes estimate) for the 4 middle-out models incorporating the PBPK-derived clearance maturation function. Run 0: no re-estimation of parameters, run 1: re-estimation of NLME-derived PK parameters, run 2: re-estimation of NLME-derived PK parameters, run 2: re-estimation of PBPK-derived clearance maturation function, run 3: re-estimation of all parameters. The dotted line depicts the clearance for a typical individual (i.e. without variability).

**FIGURE 6:**
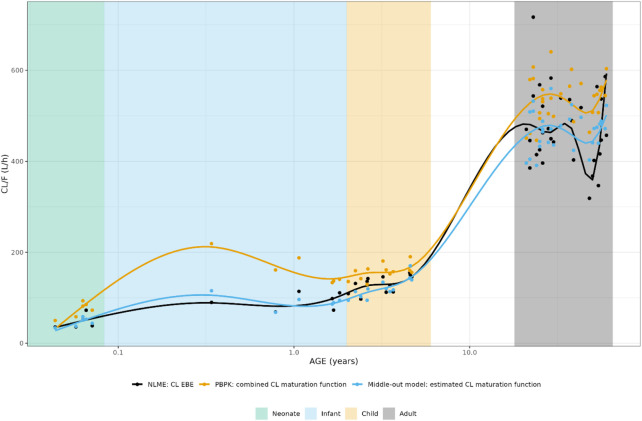
Comparison of individual apparent hydrocortisone clearance for neonates, infants, children and adults after administration of a pediatric formulation of cortisol using the NLME, PBPK or middle-out model (run 2). The closed circles and the lines represent the individual values and Loess smoothers through them. Age shown on a logarithmic scale.

In Figure 5, the individual variance estimates for CL are shown as a function of age group. Here, the distribution of the empirical Bayes estimates per individual in order to describe the observed data *post hoc* is shown. The centering of these distributions around 0 indicates an unbiased CL estimation. Inclusion of the maturation function without re-estimation induces an age-dependent bias in the CL estimation resulting in a skewed distribution of these individual estimates, which is only resolved by estimation of the maturation function parameters. In Figure 6, the CL maturation function based on only the PBPK model, only the NLME model and the estimated maturation function within the NLME framework are shown. Here it can be seen that estimation of the maturation function given the clinical datasets approaches the individual CL estimates as a function of age as fitted by the NLME model without maturation function.

#### 3.2.2 Implementation of CBG maturation

In Table 2, the parameter estimates of the implemented CBG maturation functions within the NLME model are show. Inclusion of the maturation function without re-estimation of any parameter but including fitting of the individual profiles using empirical Bayes estimates (option MAXEVAL = 0 in NONMEM) resulted in a significantly better fit as indicated by the OFV (ΔOFV = −26.85 < 15.36). Estimation of the PK parameters while keeping the maturation function constant also resulted in a better fit (ΔOFV = −95.51 < 15.36) but in an unstable model as indicated by the very large condition number (1.19 * 10^8^ > 1000) and the imprecision of some of the IIV-related parameters becoming high (IIV CL = 72.5% > 50%). Estimating the parameters of the maturation function alone or together with the PK parameters resulted in a significant improvement of the fit (ΔOFV = −53.1 < −15.36 for run 2 and −133.96 < −15.36 for run 3).

**TABLE 2:** Parameter estimates, objective function value (OFV) and condition number (CN) from the middle-out models exploring the inclusion of a maturation function for cortisol binding globulin (CBG).

Model	Base model	CBG-maturation, no re-estimation	CBG-maturation, PK-estimation	CBG-maturation, MAT-estimation	CBG-maturation, re-estimation all
Parameter	Value (rse, %)	Value (rse, %)	Value (rse, %)	Value (rse, %)	Value (rse, %)
CL [L/h]	410 (8.1)	409 (-)	612 (1.90)	409 (-)	995 (13.3)
V2 [L]	10.6 (9.4)	10.6 (-)	10.4 (12.3)	10.6 (-)	13.6 (9.27)
Q [L/h]	160 (17.9)	160 (-)	147 (21.5)	160 (-)	422 (22.0)
V3 [L]	124 (16.3)	124 (-)	122 (24.4)	124 (-)	348 (18.4)
Km [nmol]	4,810 (21.2)	4,810 (-)	5,190 (1.66)	4,810 (-)	8,820 (25.1)
Vmax [nmol/h]	21,600 (11.0)	21,600 (-)	21,500 (0.03)	21,600 (-)	32,800 (14.9)
BASEAdult	15.4 (6.33)	15.4 (-)	15.4 (6.38)	15.4 (-)	15.6 (6.54)
BASEChild	13.3 (1.94)	13.3 (-)	13.3 (1.27)	13.3 (-)	13.3 (2.82)
IIVCL (CV%)	19.2 (17.8)	19.2 (-)	22.9 (72.5)	23.2 (18.9)	22.4 (19.0)
IIVKm (CV%)	45.6 (36.3)	45.6 (-)	40.7 (51.5)	51.3 (24.0)	46.4 (33.7)
IIV_Vmax_ (CV%)	43.7 (16.7)	43.7 (-)	42.9 (23.8)	42.9 (14.5)	40.0 (17.8)
IIV_BASE_ (CV%)	33.5 (22.3)	33.5 (-)	33.5 (22.0)	33.6 (22.4)	33.6 (22.2)
IIV_BIO_ (CV%)	34.9 (19.3)	34.9 (-)	36.5 (20.4)	42.1 (18.4)	31.9 (17.2)
BASE_CBG_		0.20 (-)	0.05 (-)	0.456 (23.3)	1.83 (26.0)
MAX_CBG_		0.99 (-)	14.8 (-)	0.643 (8.29)	0.71 (36.0)
TM_50,CBG_		2.33 (-)	1.17 (-)	0.76 (113)	0.0233 0)
HILL_CBG_		0.35 (-)	0.17 (-)	0.142 (251)	0.191 (45.8)
Residual variability (CV%)	14.5 (8.0)	14.5 (-)	14.5 (8.0)	14.2 (7.32)	14.0 (7.71)
CN	155.6	NA	1.19 * 10^8^	217.5	846.6
OFV	-3,907.90	-3,934.75	-4,003.41	-3,961.00	-4,041.86

CL: Apparent clearance, V2: apparent central volume of distribution, Q: apparent intercompartmental clearance, V3: peripheral volume of distribution, K_m_: amount in depot compartment resulting in half of V_max_, V_max_: maximum absorption rate, BASE_Adult_: cortisol baseline of dexamethasone suppressed healthy adults, BASE_Child_: cortisol baseline of children with baseline measurement BLOQ, IIV: interindividual variability, BASE_CBG_: CBG, concentration at birth, MAX_CBG_: maximum CBG, concentration, TM_50,CBG_: age at which half of MAX_CBG_, is reached; HILL_CBG_, hill factor for the CBG, ontogeny function. Parameters were allometrically scaled using a body weight of 70 kg and residual variability was estimated as additive error on a log scale.

**FIGURE 7:**
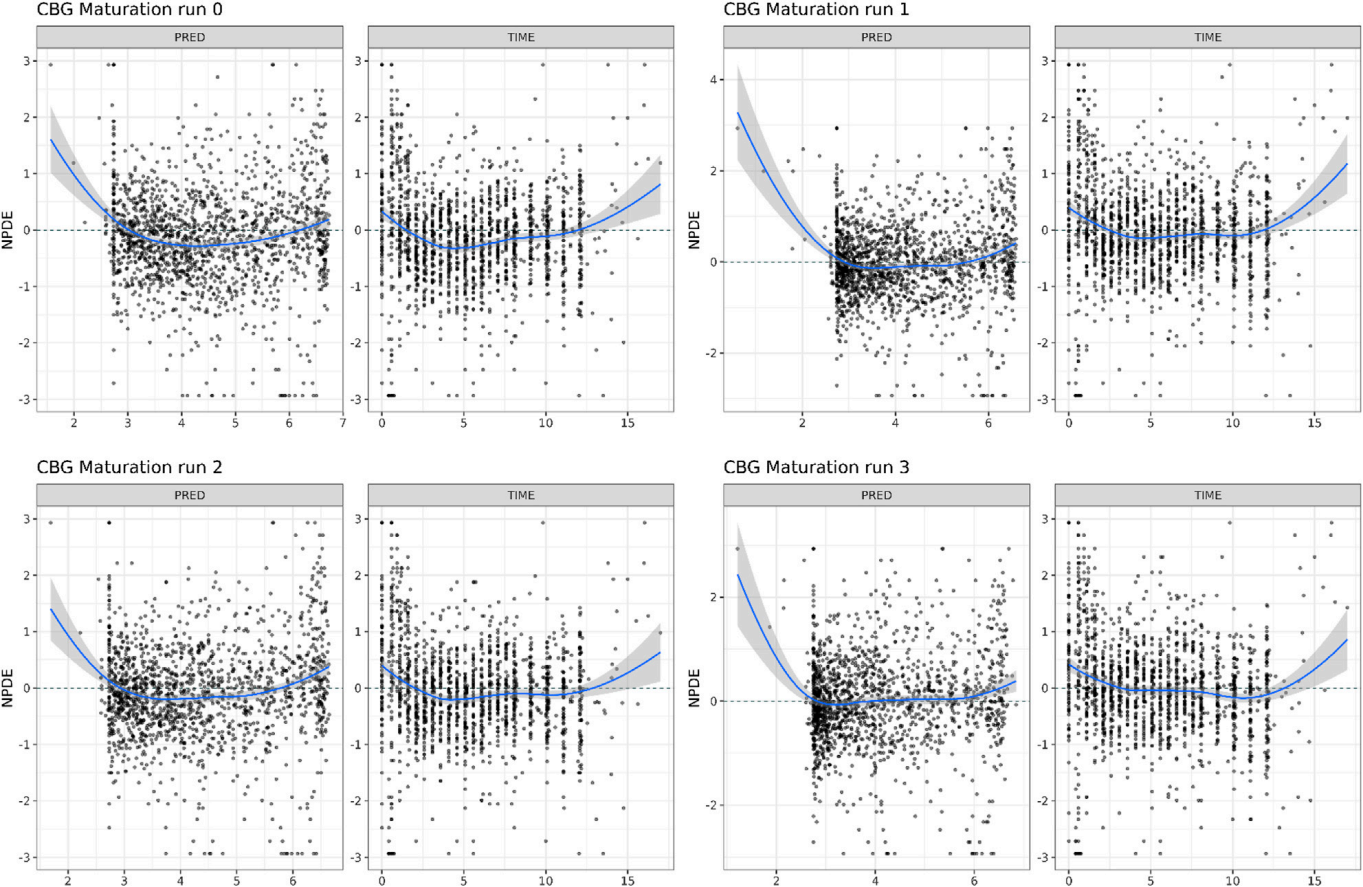
Normalized Distribution Prediction Errors (NPDE) of the 4 middle-out models incorporating the PBPK-derived cortisol-binding globulin (CBG) maturation function. Run 0: no re-estimation of parameters, run 1: re-estimation of NLME-derived PK parameters, run 2: re-estimation of PBPK-derived CBG maturation function, run 3: re-estimation of all parameters.

**FIGURE 8:**
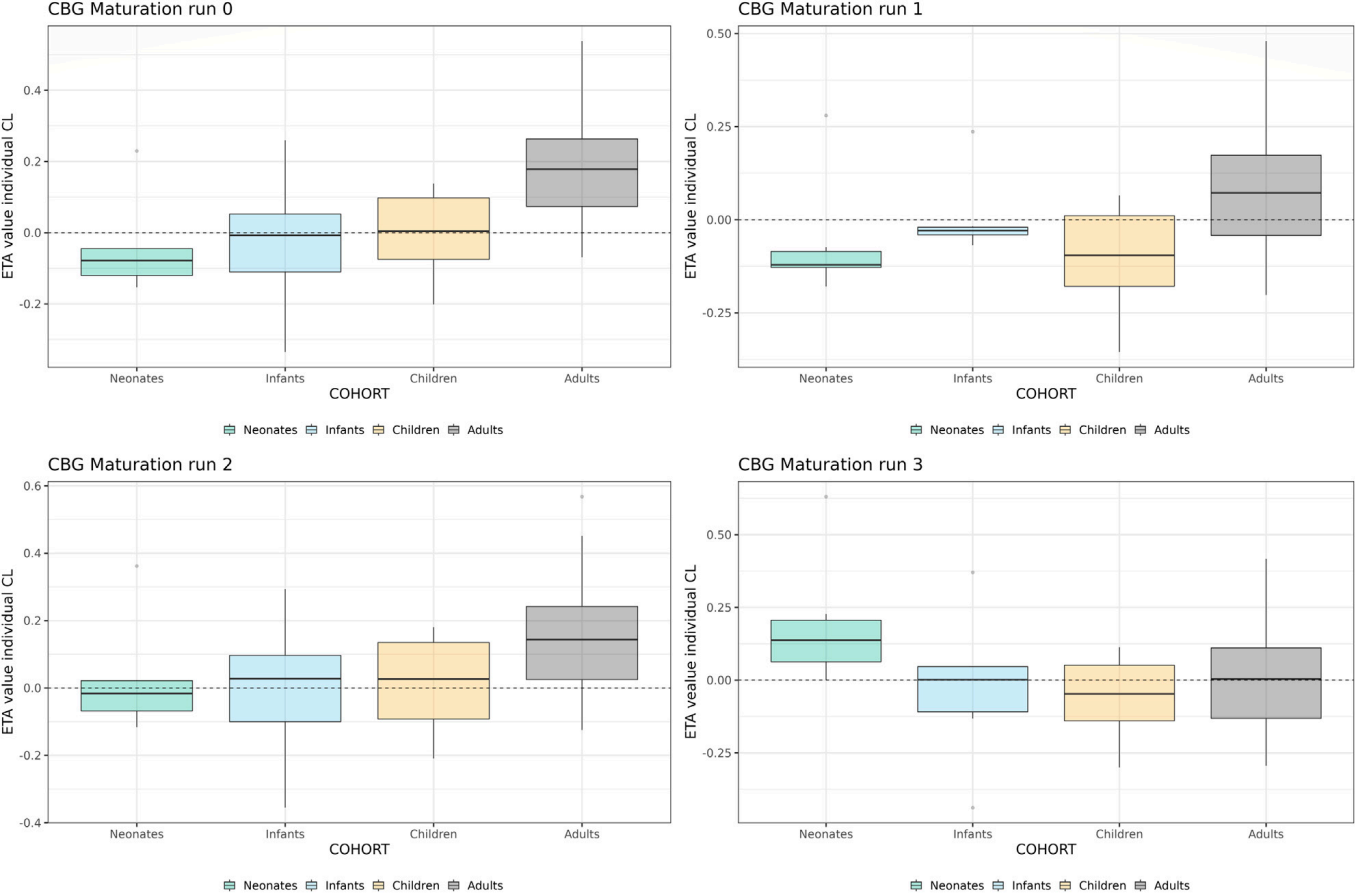
Individual clearance parameter estimates (empirical Bayes estimate) for the 4 middle-out models incorporating the PBPK-derived cortisol-binding globulin (CBG) maturation function. Run 0: no re-estimation of parameters, run 1: re-estimation of NLME-derived PK parameters, run 2: re-estimation of NLME-derived PK parameters, run 2: re-estimation of PBPK-derived CBG maturation function, run 3: re-estimation of all parameters. The dotted line depicts the clearance for a typical individual (i.e. without variability).

**FIGURE 9:**
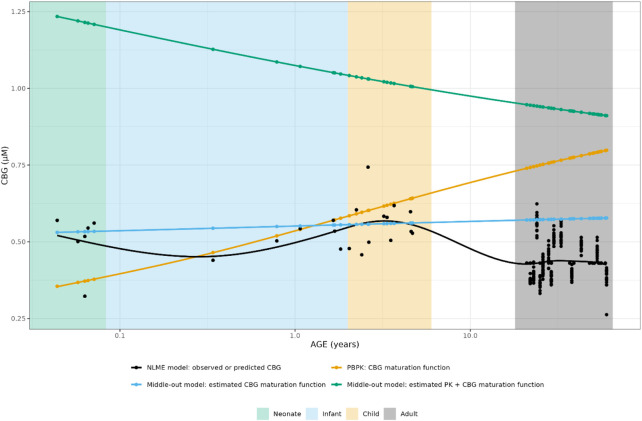
Comparison of individual cortisol-binding globulin concentrations for neonates, infants, children and adults using the NLME, PBPK or middle-out model (CBG-run 2 and run 3). The closed circles and the lines represent the individual values and Loess smoothers through them. Age shown on a logarithmic scale.

As indicated by the NPDE distributions in Figure 7 and the individual variance estimates for CL in Figure 8, the inclusion of a maturation function for CBG results in a biased estimate of the neonatal PK. Estimation of the maturation function alone resolves this bias at the cost of a biased adult CL. As can be seen in Figure 9, estimation of the maturation function alone using the clinical data approaches moves the CBG maturation function towards the individual CBG estimates as a function of age as fitted by the NLME model without maturation function. Estimation of all parameters simultaneously leads to a better-fitting stable model, using a strongly deviating maturation function (Figure 9) and CL-estimate (Table 2), and an underprediction of neonatal CL (Figure 8).

### 3.3 Implementation of best-performing maturation functions into NLME model

Ultimately, the inclusion of CL and/or CBG PBPK-derived maturation functions resulted in similar, or worse, model performance and the re-estimation of parameters most often led to unstable models or unrealistic parameter estimates.

## 4 Discussion

In this work, the maturation of different processes contributing to the PK of hydrocortisone were investigated using a middle-out approach, combining insights from clinical data analyzed using NLME modelling with ontogeny data from a PBPK model. As a first step, the conclusions of both techniques were compared to each other, showing significant differences between the two approaches. Indeed, the maturation of hydrocortisone CL was predicted differently between the two models for children between 1 month and 1 year of age but similar for the rest of the pediatric age range. For the maturation of CBG, NLME approaches predicted that no significant maturation takes place over the entire pediatric age range (Melin et al., 2017; Melin et al., 2019) while a PBPK approach showed an increase in CBG concentration from birth to adulthood (Bonner et al., 2021).

The insights from PBPK, based on an extensive review of the literature sources available at the time, were implemented in an NLME framework based on the model fitted to clinical data (Melin et al., 2017; Michelet et al., 2020) and interrogated for their potential to describe hydrocortisone PK data over the pediatric age range. Inclusion of the CL maturation function did not result in a significantly better description of the clinical data, and re-estimation of the maturation function parameters was not supported by the data. For the maturation of CBG, a better description of the clinical data was suggested by the fit, but only when the maturation function was either 1) re-estimated to approximate a stable CBG concentration over the entire age range or 2) estimated to be a decreasing function from birth on combined with a deviating PK model. Furthermore, large differences in parameter values were observed between the different re-estimation steps, indicating a discrepancy between the modeling approaches or their underlying data.

As neither the CL nor the CBG maturation function could show convincing improvements in the description of the clinical pediatric dataset, their implementation together in an optimal middle-out model was not successful. Several reasons can be proposed for this mismatch between the NLME and PBPK approach. First, a PBPK model includes maturational processes in a mechanistic way, modulating only the processes which are governed by the enzyme of which the maturation is considered. An NLME model, in contrast, lumps processes together into empirical compartments which consists of an arbitrary number of the abovementioned processes. A straightforward example of this is the maturation of the first-pass effect and bioavailability, which would be considered in a carefully constructed PBPK model, but is not taken into account in the maturation function of apparent CL (CL/F) in the NLME/middle-out approach. Mechanistic investigation of the processes governing the first pass processes of hydrocortisone PK would considerably help to elucidate the maturation of bioavailability and absorption of hydrocortisone in the pediatric population. In the current study, all data was derived from individualized hydrocortisone granules which are immediate release. However, a modified-release formulation would have a more profound effect on bioavailability and absorption, which would need to be characterized in order to update the underlying structural PK model.

Second, the current findings are dependent on the nature of clinical data which is available for hydrocortisone PK in CAH pediatric patients. Our current clinical dataset is collected from clinical trials, where different cohorts were selected, for regulatory and ethical reasons, based on distinct age cut-offs. These cohorts were defined as neonates (0–1 month), infants (1 month–2 years) and children (2–6 years). In general it is difficult to recruit such young children, especially for a rare disease, into the clinical trials and hence the overall numbers of pediatric patients is low. This is further compounded by, when children are recruited into the trial, they are often recruited towards the upper end of these age groups–this is particularly evident for the infant cohort where there are more patients towards 2 years of age, resulting in a lack of data between 1 month and 12 months of age. This makes the discrepancies between the PBPK and NLME approach challenging to validate with the current dataset, because there is a large gap in the data where, potentially, the most scientific interest lies in the maturation functions of enzymes with early age. 

Although the implementation of the PPBK-derived maturation functions into the NLME framework show potential for better description of the PK of hydrocortisone in children, clinical data available to date do not support them formally. Thus, more PK data in young infants would be very beneficial to further develop and refine these modelling approaches. This sparsity of infant data also puts into question the typical staggered approach of pediatric clinical trials (although it is acknowledged that this needs to be balanced by the regulatory and ethical requirements of running the clinical trial with pediatric patients), as these age cut-offs will more likely recruit older children per cohort (Manolis et al., 2011). Indeed, our new insigh can contribute to the concrete design of next clinical trial.

Third, the maturation functions derived in the PBPK framework also contain uncertainty. Both the 11-βHSD2 and 5α-reductase maturation functions were derived from data on the ratio of urinary concentrations, which might need to be corrected for the renal function relative to adults. This correction was applied before to quantify the maturation of CYP2D6 and CYP3A4 in the first year of life (Johnson et al., 2008). Refitting the maturation functions for the metabolic enzymes on the metabolic ratios considering relative renal function might be a first step towards closing the gap between the two approaches depicted in this work. The maturation function for CBG was fitted on very variable data, with a lack of data for children over 12 months of age, indicating the need for further confirmation of this maturation function.

In this investigation the prior information of the PBPK-derived maturation function was either taken at value or re-estimated, i.e., it was taken as an uninformative or informative prior. Given more information about the relevant age ranges as described above, Bayesian approaches could be applied to explore the space of models in between the extreme solutions presented in this work. Furthermore, the impact of the explored models on dosing recommendations was outside the scope of this work, but could be explored once the gap between the two approaches depicted in this work is closed. Indeed, an adequate description of HC interindividual variability as a function of age would directly impact personalized dosing, moving from body-weight based dosing to age- and body-weight dosing (Melin et al., 2020).

## 5 Conclusion

The maturation of different PK processes impacts the treatment of pediatric CAH patients with hydrocortisone. In current population NLME PK and PK/PD models, often only body weight is considered as covariate to explain the impact of age on hydrocortisone PK. In this work, insights of a PBPK modelling approach into maturation of hydrocortisone CL *via* 5α-reductase and 11-βHSD2 and cortisol binding *via* CBG were introduced in a NLME model fitted to pediatric clinical data. The discrepancies between the approaches show the importance of applying multidisciplinary methodologies in the analysis of pediatric data and of the balanced collection of clinical data across the pediatric age range. Lastly, further investigation of the maturation of 5α-reductase and 11-βHSD2 between 1 month and 12 months of age, and the maturation of CBG across the entire age range, is warranted for further development of these modelling approaches.

## Data Availability

The data analyzed in this study is subject to the following licenses/restrictions: Sharing of the clinical data analyzed in this work can be discussed upon reasonable request to the authors. Requests to access these datasets should be directed to r.j.ross@sheffield.ac.uk.
